# From Womb to Mind: Prenatal Epigenetic Influences on Mental Health Disorders

**DOI:** 10.3390/ijms26136096

**Published:** 2025-06-25

**Authors:** Diana Álvarez-Mejía, Jose A. Rodas, Jose E. Leon-Rojas

**Affiliations:** 1 Cerebro, Emoción y Conducta (CEC) Research Group, Escuela de Medicina, Universidad de las Américas (UDLA), Quito 170125, Ecuador; dianaluceli.alvarez@udla.edu.ec; 2 School of Psychology, University College Dublin, D04 V1W8 Dublin, Ireland; 3 Escuela de Psicología, Universidad Espíritu Santo, Samborondón 092301, Ecuador

**Keywords:** epigenetics, neurodevelopment, mental health, psychiatry, prenatal, DNA methylation, histones

## Abstract

The intrauterine environment is increasingly recognised as a critical period for the emergence of mental health vulnerabilities. This review explores how adverse maternal exposures, such as psychological stress, infection, malnutrition, and environmental toxins, can disrupt foetal neurodevelopment via epigenetic mechanisms, contributing to the risk of psychiatric and neurodevelopmental disorders. Focusing primarily on human studies, we synthesise evidence on DNA methylation, histone modifications, and non-coding RNAs as key pathways through which the intrauterine environment influences gene regulation in the developing brain. We examine how timing of exposure, foetal sex, and gene–environment interactions modulate these effects, with particular attention to disorders such as schizophrenia, autism spectrum disorder, depression, and anxiety. The placenta emerges as a central mediator, both reflecting and shaping epigenetic changes in response to maternal signals. We also discuss the reversibility of epigenetic marks and highlight emerging interventions, including nutritional supplementation and maternal mental health support, that may buffer or reverse prenatal epigenetic programming. Methodological challenges are addressed, including tissue specificity and causal inference, and future directions are proposed toward integrating epigenetic biomarkers into early risk assessment and precision mental health and psychiatry. This review emphasises the importance of the prenatal period as a window of vulnerability and opportunity for shaping lifelong mental health.

## 1. Introduction

It is increasingly recognised that the intrauterine environment can shape neurodevelopment in lasting ways, predisposing individuals to mental health disorders later in life [1,2]. This concept aligns with the Developmental Origins of Health and Disease (DOHaD) framework, wherein adverse prenatal exposures can “program” the developing foetus with enduring consequences [3]. Early epidemiological studies established links between maternal conditions in pregnancy and elevated risks of schizophrenia, autism, and affective disorders in the offspring [4,5,6]. For example, maternal famine during World War II and maternal influenza epidemics were each found to substantially raise the incidence of schizophrenia in the children exposed in utero [7]. Such observations suggest that factors in the prenatal environment can leave biological “memories” that alter brain development and later behaviour.

Epigenetic modifications are now thought to be key mechanisms translating prenatal exposures into these long-term neurodevelopmental outcomes [8,9]. Epigenetics refers to reversible chemical changes to DNA or associated proteins that regulate gene activity without altering the DNA sequence itself [10]. Crucially, epigenetic phenomena such as DNA methylation, histone tail modifications, and non-coding RNAs can be influenced by environmental conditions during critical intrauterine developmental windows [11]. These phenomena help control when and where genes are expressed during foetal brain development, thereby shaping the formation of neural circuits. Adverse maternal factors, including stress hormones, inflammatory cytokines, nutritional deficiencies, or toxins, can perturb the normal establishment of the foetal epigenome, potentially leading to dysregulated gene expression in the brain [12]. Over the past decade, numerous human studies have begun uncovering signatures of prenatal epigenetic disruption associated with later psychiatric outcomes, suggesting epigenetic mechanisms as a biological bridge “from womb to mind.”

This narrative review was conducted following the Scale for the Assessment of Narrative Review Articles (SANRA) recommendations [13]. We queried Scopus, PubMed and the Virtual Health Library with a combination of the following key terms: prenatal, epigenetics, DNA methylation, histone modification, non-coding RNA, placenta, neurodevelopment, mental health, schizophrenia, autism, depression, and anxiety. We prioritised original human studies, systematic reviews, and high-quality meta-analyses published in the last 10–15 years, while also including older landmark studies when foundational to the field or when representing the best available evidence to date; animal studies were also included for mechanistic clarity when human studies were lacking this information or when animal studies provided a more robust mechanistic explanation than current clinical human evidence. Reference lists of key articles were manually screened to identify additional relevant sources.

Our review synthesises current advances in understanding how prenatal epigenetic modifications mediate the links between early-life exposures and mental health disorders. We focus on evidence from human studies (with the inclusion of animal studies for mechanistic clarity) and examine (1) the main prenatal epigenetic mechanisms (DNA methylation, histone modifications, and non-coding RNAs); (2) maternal influences, including stress, infection, nutrition, and toxins, that induce lasting epigenetic changes; (3) the importance of timing and critical windows of exposure; (4) specific epigenetic findings across disorders such as schizophrenia, autism spectrum disorder (ASD), depression, and anxiety; (5) interactions between genetic susceptibility and epigenetic plasticity; (6) emerging research on the reversibility of epigenetic marks and potential early interventions; and (7) methodological challenges and future directions, including integration of prenatal epigenetic insights into precision psychiatry. Throughout, we aim to provide a critical and comprehensive overview of how prenatal epigenetic programming contributes to psychiatric vulnerability, and how this knowledge might inform prevention or intervention strategies. While prior reviews have addressed prenatal epigenetics, our work offers a distinctive synthesis by focusing primarily on human evidence, integrating findings across multiple psychiatric disorders, and highlighting recent advances such as placental methylation risk loci, intervention strategies, and precision psychiatry applications. We also distinguish between human and animal studies throughout and provide structured overviews of epigenetic mechanisms and exposures for greater translational clarity.

## 2. Prenatal Epigenetic Mechanisms

**Figure 1:**
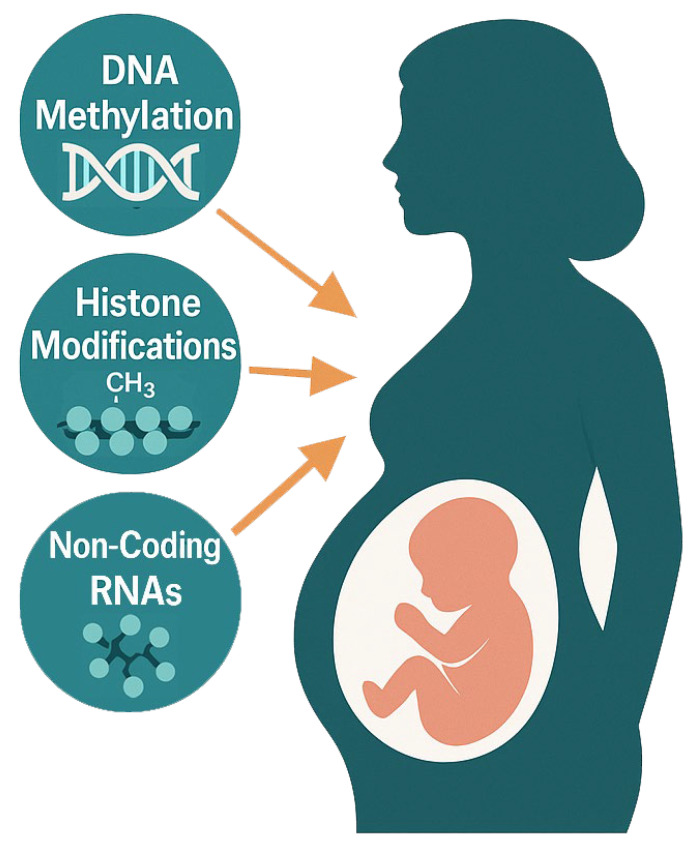
Main mechanisms of prenatal epigenetic changes.

Epigenetic regulation is central to normal brain development, guiding cell differentiation and the spatiotemporal patterns of gene expression in the foetal nervous system [14]. The three major epigenetic mechanisms are DNA methylation, histone modifications, and non-coding RNAs, all of which can be influenced by prenatal environmental conditions [15,16,17]. This section briefly outlines these mechanisms (Figure 1) and their roles in developmental gene regulation.

### 2.1. DNA Methylation

DNA methylation involves the addition of a methyl group (–CH_3_) to DNA, typically at cytosine bases in CpG dinucleotides [18]. High levels of DNA methylation in gene promoter regions often lead to transcriptional silencing of the associated gene [19]. DNA methylation patterns are normally reprogrammed during early embryogenesis and then refined during foetal development to establish cell type-specific gene expression [20]. Because DNA methylation is relatively stable, environmental insults during pregnancy can imprint long-lasting methylation changes that persist into postnatal life [21]. Indeed, the first direct evidence of prenatal environmental epigenetic programming in humans came from individuals prenatally exposed to the Dutch Hunger Winter famine of 1944–45; six decades later, these adults showed significantly reduced methylation of the insulin-like growth factor 2 gene (IGF-2) compared to their unexposed siblings [22]. Notably, this epigenetic change was specific to those exposed to famine in early gestation (periconception), highlighting how timing of exposure is crucial [23]. DNA methylation alterations in other genes have since been linked to various prenatal exposures, often detected in accessible tissues like cord blood or placenta. For example, maternal smoking during pregnancy leaves a broad DNA methylation signature on the new-born genome, with over 6000 CpG sites showing differential methylation in cord blood in infants of smokers versus non-smokers [24]. Many of these loci persist into childhood, illustrating how prenatal exposures can induce durable methylation shifts that potentially influence childhood development. DNA methylation is the most studied epigenetic marker in human prenatal cohorts due to the feasibility of measuring it in cord blood or placenta at birth [25]; thus, much of the evidence for prenatal epigenetic influences on mental health has centred on methylation differences associated with maternal exposures.

### 2.2. Histone Modifications

In the cell nucleus, DNA is wrapped around histone proteins to form chromatin [26]. Chemical modifications of histone tails (such as acetylation, methylation, phosphorylation, and others) can loosen or tighten chromatin structure, thereby regulating gene accessibility and transcription [27]. For instance, histone acetylation generally relaxes chromatin and promotes gene expression, whereas certain histone methylation marks can either activate or repress transcription depending on the residue and context [28]. These histone marks are dynamically written and erased by enzyme complexes, and they play a pivotal role in orchestrating gene expression cascades during brain development [28]. Prenatal environmental factors can perturb histone-modifying enzymes or co-factors. While direct evidence in humans is limited due to tissue access constraints, rodent and other animal models have demonstrated that prenatal stress or toxin exposures can alter histone modification patterns at genes important for neurodevelopment [29]. For example, prenatal exposure to the anti-epileptic drug valproic acid (a known histone deacetylase inhibitor) in rodents induces hyperacetylation of histones and aberrant expression of developmental genes, leading to autism-like neurobehavioral changes, a finding that parallels that of the increased autism risk seen in children of women who took valproate during pregnancy [30]. There is emerging evidence from human placenta studies suggesting that maternal conditions (like stress or high body mass index (BMI)) are associated with changes in placental histones at genes regulating foetal growth and neuroendocrine function [25]. Although large-scale human data on prenatal histone modifications are still lacking, it is biologically plausible that this mechanism contributes to how prenatal exposures exert lasting effects on gene regulation in the brain.

### 2.3. Non-Coding RNAs

Non-coding RNAs (ncRNAs), including microRNAs (miRNAs), long non-coding RNAs (lncRNAs), and others, represent a further layer of epigenetic regulation. These RNA molecules do not code for proteins but can regulate gene expression post-transcriptionally or even influence chromatin structure [17]. MicroRNAs, typically ~22 nucleotides long, bind to mRNA transcripts to degrade them or inhibit their translation, thereby fine-tuning the output of protein-coding genes [31]. Many miRNAs are expressed in the developing brain and are crucial for processes like neural differentiation and synapse formation [32]. Prenatal environmental stresses can alter the expression profile of miRNAs in both placenta and foetus. For instance, maternal stress hormones have been shown to change placental miRNA levels that target foetal neurodevelopmental genes [33]. Likewise, lncRNAs can modulate gene expression by interacting with chromatin or transcriptional machinery, and dysregulation of lncRNAs has also been implicated in neurodevelopmental disorders [34]. Although human data are still emerging, one study found that placentas from pregnancies affected by maternal anxiety had differential expression of multiple miRNAs and lncRNAs compared to low-stress pregnancies, suggesting that maternal psychological state is reflected in the ncRNA milieu at the maternal–foetal interface [35]. These changes in ncRNAs could potentially alter signalling between the placenta and foetal brain. In summary, non-coding RNAs provide an additional, plastic route through which prenatal environments may influence gene regulatory networks during brain development. Future research using transcriptomic profiling of placenta and foetal tissues is expected to shed more light on the role of ncRNAs as epigenetic mediators of prenatal risk factors.

In combination, DNA methylation, histone modifications, and non-coding RNAs form an interconnected epigenetic landscape that governs the developmental trajectory of the brain. Importantly, these mechanisms are highly sensitive to environmental cues during critical periods of gestation, when the foetal epigenome is being established. Next, we examine how specific maternal influences, ranging from psychological stress to infection and nutrition, can disturb these epigenetic processes, with consequences for the offspring’s neurodevelopment and mental health; a summary of key exposures, affected tissues and epigenetic mechanisms is provided in Table 1.

## 3. Maternal Influences on the Foetal Epigenome

**Figure 2:**
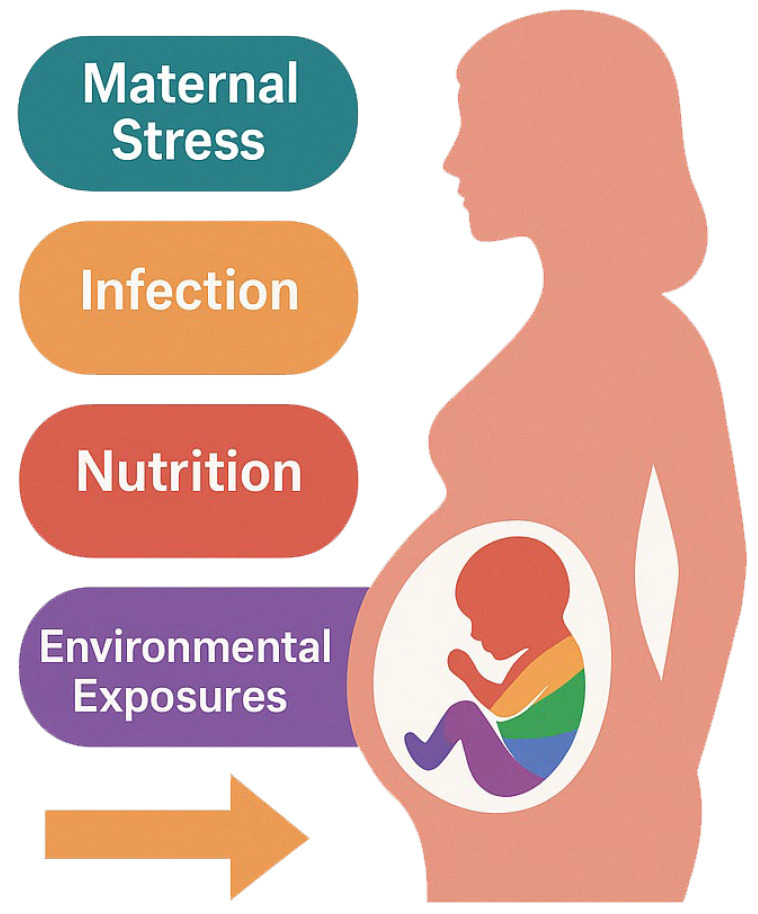
Maternal influences on the foetal epigenome.

Multiple maternal factors during pregnancy have been linked to altered epigenetic marks in offspring. Here we focus on four broad categories of prenatal influences: maternal stress, maternal infection, maternal nutrition, and environmental toxins. Each of these can perturb the intrauterine environment (through changes in hormones, immune factors, or nutrient delivery), triggering epigenetic modifications in foetal or placental tissues [35]. Such epigenetic changes are hypothesised to mediate the observed associations between these prenatal exposures and heightened risks of psychiatric or neurodevelopmental disorders in the child. Figure 2 shows the main maternal influences reported in the literature.

### 3.1. Maternal Stress

Pregnancy is a period of heightened vulnerability to the effects of maternal psychosocial stress. Epidemiological studies have long shown that offspring of women who experience severe stressors or anxiety during pregnancy are at elevated risk for a range of neuropsychiatric outcomes. For example, a large Danish cohort study found that children born to mothers who endured significant adverse life events (like the death of a close relative) while pregnant had a ~2-fold higher risk of schizophrenia in adulthood [4]. Timing appeared important in that study, as exposure to maternal bereavement stress in the first trimester conferred the greatest schizophrenia risk increase [4]. Similarly, the longitudinal Avon study in the UK reported that high maternal anxiety during gestation predicted increased behavioural and emotional problems (e.g., anxiety and ADHD symptoms) in children at 4 years old, even after controlling postnatal stress [44]. Notably, this effect was observed in both boys and girls (odds ratios ~1.5–2.1), consistent with a direct influence of antenatal maternal mood on foetal brain development [44].

Epigenetic mechanisms provide a biological framework for how maternal stress can become “embedded” in the developing foetus. Stress during pregnancy leads to elevated levels of glucocorticoids (cortisol) and other neuroendocrine changes that can cross the placenta [45]. These in turn can modify the foetal epigenome, especially in genes regulating the hypothalamic–pituitary–adrenal (HPA) axis and stress response [33]. One well-known human example involves the NR3C1 gene, which encodes the glucocorticoid receptor; in a study of mothers with mood disorders, higher third-trimester maternal depression/anxiety was associated with increased DNA methylation of the NR3C1 promoter in their new-borns’ cord blood [36]. The specific CpG site methylation in NR3C1 correlated with blunted gene expression and was functionally linked to heightened cortisol reactivity in those infants at 3 months of age. This finding suggests that maternal stress can epigenetically prime the infant’s HPA axis toward greater stress sensitivity, which may elevate risk for anxiety or depression later in life. Indeed, a growing number of studies have linked prenatal stress to methylation changes in offspring genes related to stress regulation (e.g., NR3C1, FKBP5, SLC6A4 serotonin transporter) and to altered behavioural outcome [5,33,36].

Importantly, the placenta plays a critical mediating role in maternal stress effects on the foetal epigenome. The placenta responds to maternal cortisol and releases its own stress signals that can affect the foetus. Research by Bale and colleagues has shown that maternal stress can induce sex-specific epigenetic changes in the placenta, altering the expression of genes (such as those in the insulin-like growth factor and glucocorticoid pathways) that are crucial for brain development [46]. These placental epigenetic alterations often differ between male and female foetuses, providing insight into why prenatal stress is frequently associated with sex-biased outcomes (i.e., a higher incidence of neurodevelopmental disorders like ADHD and autism in males) [45]. For instance, one study found that maternal psychosocial stress was linked to reduced placental expression of O-linked N-acetylglucosamine transferase (OGT), an enzyme that can epigenetically regulate gene expression specifically in male placentas, and low placental OGT was associated with neurobehavioral abnormalities in the male offspring [39]. Such data highlight that maternal stress can leave an epigenetic imprint both in the placenta and foetus, with potential downstream effects on offspring neurodevelopment.

Therefore, evidence suggests that maternal stress during pregnancy is associated with an increased risk of psychiatric disorders in the offspring (including schizophrenia, autism, ADHD, anxiety, and depression), and epigenetic findings support a mechanism whereby stress-related signals modify foetal and placental regulation. The timing and severity of stress, as well as foetal sex, appear to modulate these epigenetic outcomes. However, other important factors during pregnancy such as maternal infection and immune activation can also act as a potent prenatal influence, through a similar epigenetic influence.

### 3.2. Maternal Infection and Immune Activation

Maternal infections during pregnancy, particularly those accompanied by significant inflammation or fever, have been implicated as risk factors for neurodevelopmental disorders in the child. Classic epidemiological research demonstrated that prenatal exposure to viral infections such as influenza or rubella can sharply increase the incidence of schizophrenia or autism in the exposed birth cohorts [47,48]. For example, a landmark birth cohort study using archived maternal serum provided serologic evidence that first-trimester maternal influenza infection raises the risk of schizophrenia in the adult offspring seven-fold compared to unexposed pregnancies [48]. No increased risk was seen with second- or third-trimester flu, indicating a critical early window during which maternal infection can disrupt foetal brain development. Similarly, population studies in Denmark found that maternal hospitalisation for a serious infection in the first trimester, especially viral infections, was associated with a significantly elevated risk of autism spectrum disorder (ASD) in children (adjusted hazard ratio ~2.9 for first-trimester viral infection) [49]. Second-trimester maternal bacterial infections were also linked to a more modest increase in ASD risk (aHR ~1.4) [49]. These data underscore that maternal immune activation (MIA) at certain gestational stages can have lasting neurodevelopmental consequences [50].

The biological plausibility for these associations is strong, as inflammatory molecules from the mother can cross into the foetal circulation or trigger changes in the placenta [37]. Animal models of MIA have shown that key pro-inflammatory cytokines (e.g., interleukin-6 and interleukin-1β) have been shown in animal models to alter foetal brain gene expression and microglial activation, resulting in behaviours relevant to ASD and schizophrenia in the offspring [51]. Human cohort studies, such as those using maternal serum cytokine levels or cord blood methylation, provide preliminary correlative evidence, linking maternal cytokine levels with child outcomes. One study reported that mothers of children who developed ASD with intellectual disability had elevated mid-gestational levels of certain cytokines/chemokines (IL-4, IL-5, IL-13, IL-17, CCL2) compared to controls [52]. Maternal C-reactive protein (CRP), a general inflammatory marker, measured in early pregnancy has also been associated with increased ASD risk in the offspring [52]. These findings suggest that maternal immune dysregulation can influence the developing brain.

Epigenetic modifications are a likely pathway through which maternal infection and inflammation exert their teratogenic effects. Inflammatory cytokines can activate intracellular signalling cascades that converge on epigenetic regulators, altering DNA methylation or histone acetylation at genes involved in neurodevelopment [51]. Recent research indicates that placental epigenetic changes may be a key mediator in infection-related risk. In a mouse model of maternal immune activation (MIA), pronounced changes in placental DNA methylation were observed, leading to dysregulated expression of neurotransmitter-related genes in the placenta and foetus. Notably, some of these epigenetic and gene expression changes persisted into subsequent generations of rodents, raising the possibility of transgenerational transmission of risk via epigenetic inheritance [53].

Human data on prenatal infection-related epigenetic changes are beginning to emerge. One pilot study examined new-born DNA methylation profiles in relation to maternal infections and found suggestive differences in immune and neurodevelopmental genes, though results were not yet consistent enough to identify specific robust biomarkers [54]. A study of pregnancies affected by the COVID-19 pandemic reported differential methylation of neurodevelopmental gene pathways in infants born to SARS-CoV-2-positive mothers, hinting that even intrauterine exposure to a novel virus may leave epigenetic marks [41]. However, a larger epigenome-wide analysis concluded that common maternal infections (e.g., respiratory infections not requiring hospitalisation) did not produce strong, lasting methylation changes in offspring blood [49]. This suggests that the intensity of maternal immune activation is a critical factor; severe infections with systemic inflammation are more likely to perturb the foetal epigenome than mild, localised infections. In that sense, maternal infections and the accompanying inflammation have the potential to increase the risk of disorders like schizophrenia and ASD in offspring, especially when these exposures occur during sensitive periods (e.g., early to mid-gestation) or when infections cause a strong immunological reaction [49,50]. Epigenetic disturbances, potentially in the placenta and foetal brain, are thought to underlie these effects. The placenta may act as a sensor and mediator of maternal immune signals, with infection-induced placental dysfunction or epigenetic changes leading to suboptimal support for foetal neural development [39]. Future research integrating immunological and epigenomic data from maternal, placental, and foetal tissues will further clarify how infections reshape the developmental trajectory.

### 3.3. Maternal Nutrition

Nutritional factors in pregnancy profoundly influence foetal development, including the brain, by providing essential substrates for growth and for epigenetic processes (like methylation). Both severe malnutrition and specific micronutrient deficiencies during gestation have been linked to greater vulnerability to psychiatric disorders in offspring. The most striking evidence comes from historical famine studies [55]. The Dutch Hunger Winter of 1944–45, a famine that struck a well-documented population, has provided a natural experiment on prenatal nutrition. Research on individuals conceived or gestated during this famine found higher rates of schizophrenia and affective disorders in adulthood compared to unexposed cohorts. In one analysis, women exposed to the famine in the first trimester had roughly twice the risk of schizophrenia, while men did not show this effect, suggesting a sex-specific and early-gestation impact [23]. Additionally, those exposed to famine in mid or late gestation showed a higher incidence of major affective disorders (such as depression) in adulthood [23]. These findings indicate that prenatal nutritional deprivation at critical periods can increase the lifetime risk of serious mental illnesses. Certainly, inadequate nutrition, particularly of methyl-donor nutrients, during early development can result in persistent changes to the epigenome [56]. Individuals prenatally exposed to famine (especially during periconception or first trimester) exhibit distinct DNA methylation differences decades later at multiple genes; famine-exposed adults had been shown to exhibit lower methylation at the IGF-2 differentially methylated region (DMR) compared to their unexposed siblings, consistent with an in utero nutritional effect on an imprinted gene critical for growth [23]. Subsequent studies have identified other genes with lasting methylation changes associated with prenatal famine exposure, many of which are involved in metabolic and neuroendocrine pathways [57]. Interestingly, these epigenetic changes may depend on the sex of the foetus and timing of exposure. Overall, the data suggest that early gestational undernutrition can “lock in” certain epigenetic states that influence developmental physiology and perhaps brain maturation, thereby contributing to disease susceptibility [55].

Specific micronutrients can also play outsized roles. Folate and choline, for instance, are crucial donors in one-carbon metabolism needed for DNA methylation [56]. Deficiencies in folate during early pregnancy are well known to cause neural tube defects, and there is some evidence linking low maternal folate to increased risks of cognitive or behavioural problems in children [58]. Choline is of particular interest for neurodevelopment and mental health as it is essential for membrane biosynthesis and is a precursor to acetylcholine; importantly, it can act as a methyl donor for DNA and histones [59]. Controlled studies have shown that extra choline supplementation in pregnant women improves certain infant brain functions (i.e., early sensory inhibition), suggesting a potential protective effect against schizophrenia-related endophenotypes [59]. This has led to the proposal that prenatal choline supplementation could serve as an early intervention to buffer the foetal brain against developmental risks. While large trials are ongoing, preliminary findings indicate that choline-supplemented infants have enhanced markers of cognitive development and stress regulation compared to controls [60]. Epigenetically, choline and folate availability can modulate global DNA methylation and specific gene methylation (such as in brain-derived neurotrophic factor (BDNF) or glucocorticoid pathway genes), potentially mediating their neurodevelopmental effects.

On the other hand, overnutrition and metabolic conditions in the mother (such as gestational diabetes or obesity) may also influence the offspring epigenome. For example, maternal obesity has been associated with altered DNA methylation in the infant’s metabolic genes and sometimes in neurodevelopmental genes, although the consequences for mental health are still being investigated [61]. One intriguing study found that higher maternal BMI was associated with an “epigenetic age acceleration” in the new-born (i.e., a discrepancy between chronological gestational age and DNA methylation-based age), hinting that the intrauterine metabolic environment can hasten developmental epigenetic aging under specific circumstances [62]. Such accelerated epigenetic aging might reflect stress on developmental processes, potentially impacting brain maturation timing. Ensuring adequate maternal intake of key nutrients (folate, choline, vitamins) during critical windows is not only vital for physical development but may also be a modifiable factor to improve mental health trajectories. Nutritional interventions are among the most promising and practical avenues for early prevention, a point we will revisit in later sections on interventions.

### 3.4. Environmental Toxins

Exposure to environmental toxins during pregnancy, such as cigarette smoke, alcohol, heavy metals, endocrine-disrupting chemicals, and pollutants, can adversely affect foetal brain development and has been associated with increased risks of behavioural and psychiatric problems [12]. Many are known to exert neuroteratogenic effects, partly through epigenetic perturbation. For instance, maternal smoking has been linked to higher risk of attention-deficit/hyperactivity disorder (ADHD) and conduct disorders in offspring, although disentangling causation is complicated by genetic and socioenvironmental confounders [63]. However, what is clear is that intrauterine tobacco smoke exposure leaves a marked epigenetic footprint on the foetus. A large meta-analysis of new-born cord blood found thousands of genomic loci with differential DNA methylation attributable to maternal smoking during pregnancy. These changes affected genes involved in neurodevelopment, hormonal signalling, and toxin metabolism, and some methylation differences (i.e., at the aryl-hydrocarbon receptor repressor gene (AHRR)) have been detected even in adolescence, indicating persistence [24]. Such methylation shifts could mediate downstream functional effects; for example, changes in AHRR and other genes might influence neurotransmitter systems or neuroimmune pathways relevant to ADHD risk. Notably, an epigenetic risk score based on the maternal smoking methylation signature in cord blood has been proposed as a marker for later health risks, underscoring the idea that prenatal toxin exposures can be captured by epigenetic biomarkers at birth [24].

Other toxins demonstrate similar epigenetic impacts. Alcohol, when consumed heavily during pregnancy (leading to foetal alcohol spectrum disorders), has been shown to alter foetal DNA methylation patterns at genes related to brain development and growth; children with foetal alcohol effects exhibit distinctive methylation changes that correlate with neurobehavioral outcomes [64]. Lead and air pollution exposure have also been studied and maternal blood lead levels have been associated with differential methylation in new-born blood at genes linked to neurodevelopment and cognitive function [65]. Likewise, prenatal air pollutant exposure (i.e., polycyclic aromatic hydrocarbons) has been tied to altered methylation of BDNF and dopaminergic genes in cord blood, which might help explain observed links between air pollution and child IQ or ADHD symptoms in epidemiological studies [66].

From a broader perspective, environmental scientists Grandjean and Landrigan compiled evidence on industrial chemicals (lead, methylmercury, polychlorinated biphenyls, etc.) causing neurodevelopmental harm and posited that epigenetic modifications are a likely mode of action for many of these toxins [42]. For example, lead exposure can inhibit DNA methyltransferases and affect histone acetylation, resulting in lasting epigenetic misregulation of genes in the brain. Endocrine disruptors (like bisphenol A or phthalates) can interfere with hormone receptors that also act as transcription factors, potentially causing epigenetic reprogramming in hormone-sensitive brain regions [42]. In animal models, prenatal exposure to these chemicals yields DNA methylation changes that correlate with altered behaviours (such as increased anxiety or impaired cognition) [42].

It is worth noting that some epigenetic changes induced by toxins may be adaptive or protective in nature; for instance, the placenta might upregulate detoxifying enzymes via epigenetic mechanisms in response to maternal smoking or pollution, as a defence for the foetus [67]. However, these compensatory changes could divert resources or have trade-offs that still impact neural development. A recent study found that DNA methylation variations in the placenta were associated with the child’s neurodevelopmental outcomes (cognitive test scores at age 4), suggesting that placental epigenetic disruptions by toxins or other exposures can be functionally linked to later brain function in the child [43].

In summary, an array of environmental toxic exposures in utero can influence the foetal epigenome, often leaving molecular scars that persist after birth. These epigenetic alterations, whether in the foetus or in supportive tissues like the placenta, likely contribute to the increased risk of behavioural and cognitive disorders observed in exposed children. The findings from maternal smoking and other exposures provide proof of concept that epigenetic changes can serve as both indicators of prenatal toxin exposure and mechanistic contributors to developmental outcomes [24,42]. This knowledge reinforces public health imperatives to minimise toxic exposures during pregnancy and opens the door to the possibility of using epigenetic biomarkers for early identification of at-risk infants.

## 4. Timing of Exposure and Critical Windows

The impact of prenatal exposures on neurodevelopment and epigenetic programming is highly dependent on when during gestation the exposure occurs. The concept of critical windows or sensitive periods is well-established in developmental biology; during certain phases of rapid growth or differentiation, the foetus is especially vulnerable to environmental perturbations [68]. In the context of mental health outcomes, mounting evidence indicates that exposures in early gestation (e.g., first trimester or around conception) often have different and sometimes more severe or pervasive effects than exposures in later trimesters [14,46]. These timing effects are closely tied to what developmental processes are occurring and how the epigenome is dynamically regulating those processes at that time.

A clear example comes from maternal infections: as noted, first-trimester influenza in mothers carried a seven-fold increase in schizophrenia risk in offspring, whereas second- or third-trimester influenza did not significantly elevate risk [48]. Early gestation is when fundamental neurodevelopmental events (neural tube formation, neuroprogenitor cell proliferation, initial brain patterning) take place [69]. It is also a period of extensive epigenetic reprogramming; the embryo’s epigenome is being reset and then re-established in tissue-specific ways. An insult like a viral infection during this phase can disrupt these epigenetic setting processes. In the influenza study, even when considering a broader early-to-mid gestation window, schizophrenia risk was increased three-fold, whereas late pregnancy exposure had no effect [48]. This suggests that early prenatal immune activation can alter developmental trajectories (potentially via long-lasting epigenetic changes in neurodevelopmental genes), whereas similar exposures later might be mitigated or less disruptive once basic brain architecture is in place.

Nutritional studies illustrate timing effects as well. The Dutch Hunger Winter findings were very specific; only those conceived during the famine (periconception/first-trimester exposure) showed significant epigenetic changes (IGF-2 hypomethylation) decades later, whereas those exposed in mid or late gestation did not show the same IGF-2 mark [57]. Likewise, schizophrenia risk was elevated for first-trimester famine exposure (notably in female offspring) but not for the second trimester in that cohort [22]. Intriguingly, affective disorders showed the opposite pattern, with famine exposure in the second/third trimester linked to higher risk of adult depression [55]. This may indicate that different critical windows correspond to different outcomes; early gestation undernutrition might affect brain developmental processes relevant to schizophrenia (i.e., cortical neuron migration, which occurs early), while mid-gestation undernutrition might affect processes more relevant to mood regulation (perhaps the development of limbic or HPA axis structures that mature slightly later) [70]. In terms of epigenetics, the periconceptional period is when the embryonic genome undergoes global demethylation and remethylation; a famine during that time can lead to systemic methylation changes. By mid-gestation, methylation is more stable globally, but targeted changes at specific gene loci (like those controlling neurotransmitter systems) can still occur in response to the environment and cause significant changes in brain function [71].

The sex of the foetus can also influence the timing of events; maternal stress or famine may preferentially impact one sex, like the famine–schizophrenia association affecting more female offspring whereas many stress-related outcomes (ADHD, autism) are more common in males [57]. One reason is that male and female foetuses differ in developmental timing and placental biology. Male foetuses often grow faster and may be more vulnerable to early stressors that perturb growth, while female foetuses might be more buffered early but affected by cumulative stress later [72]. Epigenetically, the placenta of male vs. female foetuses may show different baseline patterns and responses; male placentas tend to be more responsive (or sensitive) to cortisol and inflammatory signals with distinct gene expression and methylation changes [40]. Therefore, an exposure at a given gestational stage could cause divergent epigenetic and developmental effects in males versus females, contributing to sex-biased risks [40]. Timing and sex effects together underscore the complexity of prenatal epigenetic influences; however, more studies are needed to clearly elucidate these complex interactions.

Another important notion is that dose and duration of exposure within a window matter. Short, acute exposures (e.g., a brief high fever) might be less likely to induce stable epigenetic changes than chronic exposures (e.g., sustained maternal depression or smoking throughout pregnancy) [73]. Some epigenetic alterations require a threshold of exposure to occur. For example, mild infections that do not provoke a strong maternal immune response might not affect the foetal epigenome, whereas a severe infection with prolonged inflammation might surpass the threshold to alter epigenetic programming of neuroimmune genes. This is why, when considering prenatal influences on mental health, the timing of foetal exposure can be as crucial as what the foetus is exposed to. The first trimester (and even the periconceptional period) is often a critical window for epigenetic disturbances with long reach, given the major developmental milestones and epigenome establishment occurring then [21,45]. Middle- to late-gestation exposures can also have significant impacts, particularly on systems maturing at those times—for instance, synaptogenesis and stress-response systems in late gestation [35]. Recognising critical windows helps target preventive measures; ensuring maternal nutrition and minimising stress in early pregnancy or timing vaccinations to avoid early-pregnancy infections could reduce risk [74,75]. It could also have the potential to inform the interpretation of epigenetic biomarkers; a methylation change present at birth might indicate an exposure during a specific trimester, which can guide clinical history-taking and early interventions in the future.

## 5. Specific Mental Health Disorders and Prenatal Epigenetics

Different psychiatric and neurodevelopmental conditions may trace in part to different prenatal risk pathways and epigenetic signatures. In this section, we examine evidence linking prenatal epigenetic alterations to specific disorders: schizophrenia, autism spectrum disorders (ASD), depression, and anxiety. While these conditions certainly have multifactorial aetiologies (with genetic, postnatal, and social factors contributing), focusing on the prenatal epigenetic component sheds light on early developmental origins and potential opportunities for prevention.

### 5.1. Schizophrenia

Schizophrenia has been one of the most intensively studied disorders in the context of prenatal risk factors. The neurodevelopmental hypothesis of schizophrenia establishes that disruptions in early brain development increase the likelihood of psychosis emerging in adulthood. Prenatal exposures such as maternal malnutrition, infection, and extreme stress have all been associated with higher schizophrenia risk, as described earlier [7,22,47]. Epigenetic findings are now illuminating how these early insults might converge on common developmental pathways in the foetus.

One recurring theme is the role of the placenta in schizophrenia risk. Remarkably, recent genetic studies have found that many schizophrenia-associated gene variants show heightened expression in the placenta (especially under stress conditions), suggesting that part of the genetic risk for schizophrenia is mediated via placental function [1]. For example, a study by Ursini et al. discovered that complicated pregnancies (those with maternal hypertension, infection, etc.) combined with a high genetic risk load led to abnormal placental gene expression profiles, which in turn were linked to altered early brain development in the offspring [37]. They identified a set of genes, dubbed the “placental genomic risk”, that were expressed in placental tissue and seemed to modulate schizophrenia risk only in the presence of prenatal adversity [37]. Many of these genes are involved in immune and hormonal processes, reinforcing the idea that prenatal environmental stress acting through the placenta can trigger changes that predispose individuals to schizophrenia decades later [35].

Epigenetic modifications in the placenta may bridge genetic risk and environmental exposure. A 2025 study in *Nature Communications* provided strong evidence for this bridge; it showed that a portion of schizophrenia’s genetic risk is actually exerted through effects on placental DNA methylation [25]. By mapping foetal placental methylation quantitative trait loci (mQTLs) and integrating with psychiatric GWAS data, the researchers found that certain risk gene variants for schizophrenia (as well as bipolar disorder and major depression) influence disease susceptibility by altering methylation of specific placental genes [25]. In other words, the genetic risk may lie dormant unless it causes an epigenetic change in the placenta that then affects foetal brain development. This was supported by Mendelian randomization analyses indicating potentially causal associations between placental methylation at particular loci and schizophrenia outcomes [25]. The implicated loci were related to neurotransmission and hypoxia responses, aligning with long-held theories that prenatal hypoxic insults and abnormal neurochemical development contribute to schizophrenia [25]. Such findings marry the genetic and environmental hypotheses: the risk genes matter most when the intrauterine environment (reflected by placental epigenome) is perturbed.

Beyond the placenta, researchers have also examined epigenetic marks in the brains of individuals who later develop schizophrenia (though of course brain tissue can only be studied after illness onset). Post-mortem brain analyses reveal DNA methylation differences in schizophrenia patients versus controls at genes like REELIN and GAD1 (involved in neuronal development and GABAergic transmission) [76]. Some of these differences may originate in early development. For instance, methylation at certain interneuron gene promoters is altered in schizophrenia brains in a manner that could trace back to disrupted foetal development of those interneurons [77]. However, distinguishing prenatal-origin epigenetic changes from changes that occur during the illness or due to treatment is challenging and may confound results. Thus, indirect approaches like peripheral biomarkers have been used. One strategy has been to analyse neonatal blood spots (Guthrie cards) [78]. In one study, investigators found different methylation patterns present at birth in infants who eventually developed schizophrenia in adulthood, compared to matched controls [38]. Although findings are preliminary, they point to possible epigenetic biomarkers in neonatal blood that correlate with later schizophrenia, implying an early developmental aetiology. Notably, some of the top differentially methylated genes in those who developed schizophrenia were related to neurodevelopment and hormone signalling [38], consistent with known prenatal risk pathways.

Schizophrenia appears to have roots in prenatal epigenetic dysregulation triggered by environmental adversities and interacting with genetic susceptibility. Key evidence includes the Dutch famine studies (nutrition–epigenetic link), maternal infection studies (immune–epigenetic link), and integrative genomic–epigenomic analyses showing placental methylation as a mediator of risk [22,37,47]. Going forward, identifying specific epigenetic changes (in cord blood or placenta) that predict schizophrenia could revolutionise early risk assessment. Moreover, this knowledge emphasises the importance of prenatal care in preventing or mitigating maternal infections, stress, and malnutrition which may reduce the epigenetic insults that increase vulnerability to schizophrenia.

### 5.2. Autism Spectrum Disorders (ASDs)

ASDs are neurodevelopmental conditions often diagnosed in early childhood, characterised by social–communication deficits and repetitive behaviours [79]. Like schizophrenia, ASD has been linked to prenatal factors, particularly maternal immune activation, and metabolic conditions. One of the most consistent findings is that maternal infection with fever or sustained inflammation during pregnancy elevates autism risk in offspring [49,80]. For example, a study reported that maternal viral infection with fever, or even frequent febrile episodes, in the first half of pregnancy was associated with increased ASD incidence [81]. Maternal autoimmune disorders and high levels of certain autoantibodies have also been associated with autism in children, suggesting that intrauterine immune disturbances can affect the developing brain [81]. The mechanistic tie between maternal immune activation (MIA) and ASD has been heavily explored in animal models, which show that maternal IL-6 and IL-17 cytokines can alter foetal brain epigenetic landscapes and cause autism-like behaviours in offspring [82]. In humans, evidence of epigenetic involvement comes from studies of placenta and infant blood. A study from 2017 found that placentas from mothers who had elevated mid-gestation inflammatory markers exhibited a specific “epigenetic signature”—differential DNA methylation in genes related to synapse formation and microglial function—which was hypothesised to predispose the child to neurodevelopmental issues [51]. While that study did not follow children to diagnosis, it provided a plausible epigenetic link between maternal inflammation and foetal brain changes. Researchers have also identified ASD-associated epigenetic changes in cord blood. An epigenome-wide study found that neonates who later developed ASD had subtle differences in methylation at certain loci in cord blood DNA, including genes involved in neurodevelopment and immune regulation [83]. Although individual CpG changes did not replicate strongly across studies (possibly due to heterogeneity of ASD), there is convergence on pathways; for instance, several studies implicate methylation changes in genes governing axon guidance, Wnt signalling, and foetal brain patterning in ASD offspring [81]. These are processes that occur prenatally, supporting the idea of an intrauterine origin.

One particularly interesting line of evidence involves maternal metabolic conditions like diabetes and obesity, which are risk factors for ASD. These conditions can create an inflammatory and high-insulin uterine environment that may epigenetically reprogram the foetus [6]. A study observed that children of diabetic or obese mothers had differential methylation at birth in genes related to neural development and insulin signalling, some of which were shown to be related to the child’s later cognitive abilities [84]. This suggests a possible pathway whereby maternal metabolism influences autism risk via epigenetic modulation of neurodevelopmental genes. Autism is also notable for some well-defined genetic causes (e.g., syndromic autism in Fragile X or Rett syndrome) [85]. Even in these cases, prenatal environment can modulate severity. For example, Rett syndrome (caused by mutations in the MECP2 epigenetic regulator) shows variability possibly related to early environmental factors, meaning that even when a primary genetic lesion exists, environmental epigenetic interactions can influence outcomes [86].

Overall, while the genetic architecture of ASD is significant, a prenatal epigenetic component is evident. Large consortia like the MARBLES (Markers of Autism Risk in Babies—Learning Early Signs) study are combining maternal exposure data, placental epigenetics, and infant outcomes to pinpoint which early epigenetic markers are predictive of ASD [87,88]. The hope is that an epigenetic signature at birth could identify infants at high risk for autism, enabling earlier monitoring or interventions. Already, some researchers have proposed panels of differentially methylated regions (DMRs) in cord blood that, in retrospective analyses, distinguish children who later developed ASD [83]. These panels need validation, but they underscore the principle that autism has prenatal roots traceable in the epigenome. Current evidence suggests that ASD risk is influenced by prenatal conditions like maternal immune activation and metabolic factors, which likely exert their effects through epigenetic modifications in the developing brain and placenta [6]. Epigenetic disruptions in pathways of synaptic development, neuroimmune function, and neural patterning have been observed in association with ASD. As with schizophrenia, timing matters; studies hint that early- to mid-gestation exposures (when synapse formation and neural migration are underway) are particularly relevant for autism risk [50,68]. Recognising these links reinforces the importance of prenatal care and opens potential for epigenetic biomarkers to aid early ASD detection as well as other often-overlooked factors as showcased in the MARBLES study [87,88].

### 5.3. Depression and Anxiety

Depression and anxiety disorders have multifactorial origins, but a growing body of research suggests that susceptibility can be programmed by experiences as early as the womb. Maternal mood disorders during pregnancy, namely prenatal depression and anxiety, have been associated with an increased likelihood of children developing internalising problems, anxiety, or depression later in life [1]. For instance, maternal depression during gestation is linked to higher odds of the child having depression in adolescence, independent of maternal mood postpartum (pointing to intrauterine effects rather than solely genetic transmission or postnatal environmental influences) [33]. Likewise, excessive maternal cortisol from stress or depression in pregnancy has been tied to children’s altered stress responses and greater anxiety traits [12]. Epigenetic studies provide some of the most direct insights here. We discussed earlier the example of the hypermethylation of the NR3C1 gene, a glucocorticoid receptor gene in new-borns that has been repeatedly associated with maternal depression/anxiety during pregnancy [36]. This hypermethylation is thought to stem from foetal exposure to high cortisol levels, which can recruit chromatin-modifying enzymes to the NR3C1 promoter. The result is an epigenetically dampened HPA axis in the child, which paradoxically may lead to over-reactivity to stress (because fewer receptors are present to provide feedback inhibition) [89]. Indeed, infants with higher NR3C1 methylation due to maternal depression exhibit larger cortisol spikes under stress. Such HPA axis dysregulation is a risk factor for later anxiety and depression [89]. Another gene studied is SLC6A4, encoding the serotonin transporter. This gene has a well-known polymorphism (5-HTTLPR) associated with stress sensitivity, but it is also subject to epigenetic regulation [90]. Higher maternal anxiety has been correlated with increased SLC6A4 methylation in infant cord blood in some studies, which could reduce serotonin transport in the developing brain and has been linked to infant temperament differences [91]. Children of very anxious or depressed mothers during pregnancy have shown methylation changes in other neuroplasticity genes as well, such as BDNF (brain-derived neurotrophic factor), potentially affecting synaptic development [89].

One compelling piece of evidence for the foetal programming of depression risk comes from studies of prenatal exposure to extreme stress events (which often induce maternal depression/anxiety). Project Ice Storm in Quebec examined children in utero during a severe ice storm disaster. Decades later, those children exhibited higher rates of cognitive and language deficits, and some had elevated internalising behaviours; DNA methylation analyses in their blood revealed distinct patterns associated with the degree of maternal hardship, affecting genes related to brain and immune function [92]. While not an overt measure of depression, these findings show that even a single acute stressor can leave a broad methylation signature that correlates with behavioural outcomes many years later. However, it is important to note that prenatal exposure to maternal depression or anxiety does not guarantee a child will develop a disorder, but it may create a vulnerability. Many studies (including a 2023 systematic review of EWAS, or epigenome-wide association studies) have noted that associations between prenatal maternal depression/anxiety and infant DNA methylation, while present, are often small in effect size and not always consistent across cohorts; they found few CpG sites that were consistently associated across cohorts, as well as substantial methodological heterogeneity as contributing factors [12]. This indicates that multiple, interacting factors determine whether a child actually develops depression/anxiety. Nonetheless, even subtle epigenetic alterations (for example, slight changes in the tuning of HPA axis genes or neurotransmitter genes) could bias the stress reactivity or emotional regulation of the child in a way that, combined with later life stressors or genetic predispositions, increases the risk of a clinical disorder [21].

In adolescent and adult patients with depression, some epigenetic differences have been noted (like hypermethylation of BDNF or certain HPA-related genes in blood or brain tissue) [93]. The extent to which these reflect prenatal programming versus postnatal stress is an area of active investigation. Longitudinal birth cohorts that follow individuals from birth (with epigenetic data archived) into adulthood will be crucial to answer that. Prenatal maternal depression and anxiety have the potential to “biologically embed” in the foetus through epigenetic modifications in stress-response and emotion-regulation pathways [5]. These epigenetic marks may not directly cause childhood psychopathology but can shape the developing stress physiology and neurocircuitry in a way that predisposes the child to anxiety or mood disorders, especially if compounded by adverse experiences later. Encouragingly, this also implies that effectively treating maternal depression during pregnancy (or bolstering maternal psychosocial support) might prevent or attenuate some of these epigenetic and developmental consequences. A recent Lancet review noted that perinatal mental health interventions could improve child outcomes, possibly by normalising stress hormone levels and thus reducing epigenetic disruption in utero [94].

Across schizophrenia, ASD, and affective disorders, we see common prenatal themes: stress, infection, and poor nutrition/toxic exposures each confer risk, and epigenetic mechanisms are frequently implicated as mediators. Each disorder may involve a distinctive constellation of genes and timings; for example, schizophrenia might involve early gestational hits to cortical development, autism might involve mid-gestational immune-inflammatory hits to synapse formation, and depression/anxiety might involve HPA axis programming throughout gestation. But what unites them is the principle that the roots of adult mental health could often extend back to the womb, with epigenetic marks as traceable evidence of those early roots. This understanding motivates a more proactive approach to mental health, focusing on prevention and intervention in the prenatal period, and informs the emerging field of precision psychiatry that aims to incorporate genetic and epigenetic information into personalised risk assessment and care. More studies are required to achieve this and to support the use of epigenetic markers as potential diagnostic or prognostic tools for mental health, but the future seems promising.

## 6. Genetic Susceptibility and Epigenetic Plasticity

Genetic and epigenetic factors were once viewed as separate or even competing explanations for disease, but it is now clear that they interact intimately. Gene–environment interactions (GxE) often manifest at the level of the epigenome; that is, genetic susceptibility can influence how strongly an environmental exposure will induce epigenetic changes, and conversely environmental factors can modulate the functional effect of risk genes through epigenetic modifications [95]. In prenatal development, this interplay is particularly salient as the foetus’s genetic makeup may render certain genes more or less epigenetically responsive to maternal influences, creating individualised trajectories of risk or resilience [96]. A quintessential example is the FKBP5 gene, which regulates stress hormone feedback. A common functional polymorphism in FKBP5 (intron 2 SNP rs1360780) moderates the impact of childhood trauma on mental health [97]. Important research demonstrated that individuals with the risk allele who experienced early-life trauma showed persistent DNA demethylation at glucocorticoid response elements in FKBP5, leading to its overexpression and a disrupted stress response system; those without the risk allele did not show such epigenetic changes under trauma [98]. Although this FKBP5 study was in the context of childhood exposure (not prenatal), it illustrates the principle that an allele-specific epigenetic change mediated the gene–environment interaction and was associated with increased risk of psychopathology (e.g., PTSD and depression) in the affected individuals [98]. Analogously, in the prenatal environment, we can imagine that foetal genes involved in metabolism, neurodevelopment, or placental function might have variants that make their promoters more susceptible to methylation or chromatin changes when exposed to, say, maternal hyperglycaemia or cortisol [11].

The placenta-focused schizophrenia findings provide a concrete human example. Genetic risk variants for schizophrenia were found to have effects on placental DNA methylation (i.e., they were placental mQTLs). If a mother’s environment is benign, those variants might not lead to any adverse outcome, but if the mother is stressed or nutrient-deprived, the variant could cause an abnormal methylation state in the placenta, impairing its function, thereby elevating risk for schizophrenia in that pregnancy [25]. Essentially, the genetic risk is unmasked by environmental challenge via an epigenetic mechanism. This model has been phrased as “genetic control of sensitivity to the environment.” In the case of schizophrenia, genes related to placental resilience (or lack thereof) under stress could explain why only some high-risk individuals (those who also had prenatal stress exposure) go on to develop the illness [25]. Another example is the serotonin transporter promoter (5-HTTLPR) short allele, known to increase sensitivity to stress [91]. Some studies suggest that children with the short allele, who were also exposed to high maternal anxiety in utero, show distinct DNA methylation profiles at the SLC6A4 locus and more behavioural inhibition, whereas either factor alone (allele without stress, or stress in long-allele carriers) was not as impactful [99]. Though data is preliminary, it supports that genotype can influence the epigenetic response to the prenatal environment.

Epigenetic plasticity refers to the capacity of the epigenome to change in response to stimuli and it is at its peak prenatally, which is both a vulnerability and an opportunity [100]—vulnerability because adverse exposures can induce maladaptive epigenetic changes in a genetically susceptible foetus, as we have discussed, and opportunity because positive environmental influences or targeted interventions can normalise or even improve epigenetic changes, potentially offsetting genetic risk [100]. For example, a foetus carrying a gene variant that slightly impairs neurotrophin signalling might, under normal conditions, have suboptimal synaptic development. But if the mother takes extra choline (which can boost methylation potential and neurotrophic factors), the foetus might epigenetically upregulate compensatory pathways, mitigating the effect of the risk gene [100]. This interplay aligns with the concept of “differential susceptibility” where some genotypes make an individual more malleable to both bad and good environments; those plasticity genes could result in worse outcomes under adversity and better outcomes under enrichment [101]. Epigenetics is a plausible mediator of such differential effects. One could speculate that a child with a high-risk genotype for ADHD might, if exposed prenatally to toxins like lead, develop more severe epigenetic disruption of attention pathways and thus more severe ADHD, but if that same child had a toxin-free, successful prenatal environment, they might epigenetically compensate and exhibit minimal symptoms, and perhaps even better focus than average if some compensatory upregulation occurred.

Another dimension to consider is transgenerational epigenetics. Some epigenetic changes in germ cells can carry into the next generation, meaning ancestral exposures could affect descendants’ mental health risk [20]. While this has been shown in animal models (e.g., stress or toxin effects persisting for multiple generations via sperm/egg epigenetic changes), evidence in humans is limited and confounded [102]. However, epidemiological hints, such as grandchildren of those who experienced famine having health effects, suggest some heritable epigenetic influence. Genetic susceptibility can influence these too as certain alleles might protect the germline epigenome from being reprogrammed by the environment, whereas others might be more permeable to it.

Genes and epigenetics work hand in hand in shaping mental health trajectories from the earliest stages of life. The paradigm here is not nature versus nurture, but nature through nurture, meaning that genetic predispositions often manifest through environmentally triggered epigenetic changes [103]. This underscores why not everyone exposed to prenatal adversity develops a disorder (i.e., genetic makeup modulating sensitivity). Conversely, it explains why not all individuals with a genetic risk allele become ill thanks to a favourable early environment, and its epigenetic influence can buffer or nullify the risk. For researchers and clinicians, this interplay highlights the importance of integrating genetic screening with epigenetic and environmental assessments in the future. Eventually, one could envision risk algorithms where a foetus with certain risk genotypes might be flagged to receive extra environmental support (nutritional supplements, stress reduction) to pre-emptively counteract potential epigenetic misprogramming. The interplay of genetic susceptibility and epigenetic plasticity thus offers both insight into the biology of risk and avenues for tailored preventive strategies.

## 7. Reversibility of Epigenetic Changes and Early Interventions

One of the most hopeful aspects of epigenetics is that, unlike DNA sequence mutations, epigenetic modifications are potentially reversible [104]. They are maintained by enzymes and protein complexes that can be targeted by interventions. This plasticity means that even if adverse prenatal exposures set an epigenetic change associated with mental health risk, there may be opportunities to mitigate or even erase these changes before they solidify into pathology [100]. Certainly, evidence from animal studies shows that postnatal interventions can reverse certain epigenetic imprints of early-life stress. In the rodent model study by Weaver et al., the adult offspring of low-nurturing mothers had increased DNA methylation of the NR3C1 (glucocorticoid receptor) gene promoter in the hippocampus, leading to high stress reactivity [29]. Remarkably, when these grown rats were given a histone deacetylase (HDAC) inhibitor (which promotes a more open chromatin state), the researchers observed removal of the methylation differences between the low-nurtured and high-nurtured groups; the HDAC inhibitor treatment restored glucocorticoid receptor expression and normalised HPA stress responses in the previously low-nurtured rats [29]. This experiment was a proof of principle that even entrenched epigenetic marks from early life could be pharmacologically reversed in adulthood, with accompanying improvements in phenotype. Translating this to humans, it suggests that epigenetic changes induced by prenatal stress might also be reversible or modifiable with the right treatment, although systemic HDAC inhibitors are not presently practical for developing children due to their significant side effects [100]. Another relevant example is cross-fostering in rodents. Pups born to mothers that experienced gestational stress (i.e., epigenetically programmed for high anxiety) showed reduced anxiety behaviour and partial normalisation of gene methylation when cross-fostered at birth to very attentive dams [20]. The enriched postnatal environment (high licking/grooming) appeared to trigger active demethylation of some stress-related genes in the pups’ brains, mitigating the prenatal epigenetic programming [20]. This underscores that early interventions, even after birth, can harness developmental plasticity to counteract prenatal epigenetic scars. In humans, this might correspond to ensuring a nurturing, low-stress infancy (for instance, through parent training or enriched day-care for at-risk babies) to see if some of the at-birth epigenetic changes in risk fade over time [105].

Importantly, the degree of reversibility likely depends on the specific epigenetic change and tissue. DNA methylation changes established during early development can be very stable, persisting decades as shown in famine studies [23], but even these may gradually change with postnatal environment or targeted therapies. For example, one study found that the NR3C1 methylation differences in infants of depressed vs. non-depressed mothers diminished by childhood if the postnatal environment was supportive, implying partial natural normalisation of the epigenome over time in a better environment (the so-called “catch-up” in epigenetic age) [36]. The developing brain in childhood and adolescence still has significant epigenetic plasticity, offering a window during which interventions could reshape trajectories for better or worse [106].

Potential early interventions to mitigate prenatal epigenetic risk fall into several categories. Nutritional supplementation with nutrients like folate, choline, and zinc act as cofactors for epigenetic enzymes; supplementing at-risk pregnancies might prevent or correct detrimental epigenetic changes [107]. Folic acid fortification has already drastically reduced neural tube defects [58]. Currently, trials are examining high-dose choline supplementation in pregnant women to improve offspring brain development, and preliminary results show that infants of choline-supplemented mothers have better early information processing and a trend toward fewer attention problems [59]. Choline acts both as a methyl donor and as a neurodevelopmental stimulant (via acetylcholine receptors), so it could directly counteract some effects of maternal stress or alcohol on the foetal epigenome [59]. If these findings hold, recommending higher choline intake (e.g., through diet or supplements) during pregnancy could become a preventative strategy for schizophrenia or ADHD in high-risk populations [108]. Stress reduction and mental health support, intervening on maternal stress, depression, and anxiety during pregnancy, could not only benefit the mother but likely prevent harmful epigenetic programming in the foetus [12]. Randomised trials of cognitive–behavioural therapy or antidepressant treatment in pregnant women with depression have shown improvements in infant outcomes like easier temperament and possibly more normative cortisol patterns [109]. While foetal epigenetic markers were not always measured, it stands to reason that reducing maternal cortisol and inflammation could result in a more typical epigenetic state in the baby (for example, preventing excess NR3C1 methylation) [89]. Some ongoing studies are examining whether mindfulness programs in pregnancy can alter placental expression of stress-related genes or infant telomere length and methylation age as proxies for improved developmental outcomes [110]. Another potential intervention to mitigate risks could be pharmacological epigenetic therapies with the creation of more refined epigenetic drugs that might target specific pathways implicated in prenatal programming. For example, if a particular histone methylation on a neurotrophic gene is found to be reduced by prenatal infection, a drug that boosts that histone methylation mark (or inhibits its eraser enzyme) might be used in the early postnatal period to compensate. Currently, epigenetic drugs (like HDAC inhibitors or DNA methylation inhibitors) are used in cancer and are too blunt for use in babies [95,111]. But perhaps demethylating agents could be delivered in a targeted way (e.g., nanoparticle delivery to the placenta or brain) in the future to reverse specific changes [112]. This remains speculative, and safety is paramount, so such interventions would require extensive research and validation. Finally, early childhood enrichment can capitalise on neural plasticity to reshape epigenetic changes; for example, consistent nurturing care, responsive parenting, and cognitive stimulation can improve emotional regulation in high-risk children [113]. Studies of institutionalised children who were later placed in foster care have shown partial recovery in cognitive function and stress physiology, paralleling changes in DNA methylation trajectories relative to children who remained institutionalised [114]. Although those changes occurred postnatally, they demonstrate the capacity for the epigenome to respond beneficially to improved environments.

A fascinating emerging idea is whether we could directly monitor and adjust epigenetic changes at birth. With technologies like epigenome editing (using CRISPR-based tools to add or remove methylation at specific sites) being developed in the lab, one could imagine a scenario far in the future where a new-born’s blood epigenetic profile is assessed, and if it shows, say, extreme hypermethylation of multiple neurodevelopmental genes due to prenatal exposures, targeted epigenetic editing therapies could be applied to blood cells (or perhaps induced neural cells) to prevent mental health issues in the future [115]. This is very futuristic and not close to current clinical practice but underscores the theoretical reversibility of epigenetic states. In humans, one real-world demonstration of perinatal epigenetic intervention success is the prevention of intellectual disability in babies with congenital hypothyroidism; by promptly giving thyroid hormone at birth, the adverse epigenetic effects of low hormone on brain development are averted, resulting in normal IQ [116]. While not an epigenetic drug per se, thyroid hormone likely influences epigenetic regulation of myelination genes, and other factors of the epigenetic machinery; its timely replacement shows how early medical intervention can normalise developmental epigenetic trajectories that would otherwise veer off course.

Overall, the reversibility of epigenetic changes provides a rationale for early-life interventions as a form of preventive psychiatry. It shifts our focus from treating disorders after they manifest to modifying biological risk factors in infancy or even prenatally. This could mean ensuring every pregnancy has adequate nutrition and low stress, managing maternal illnesses effectively, and supporting families so that new-borns start life in an optimal environment. By doing so, we are in essence performing an “epigenetic therapy” at the population level by promoting the establishment of a healthy epigenome that will support mental wellness. While challenges remain in identifying which specific changes to target and how, the principle that early intervention can alter developmental trajectories is well supported by both animal and human evidence.

## 8. Methodological Challenges and Future Directions

### 8.1. Methodological Challenges and Limitations

Research on prenatal epigenetics and mental health faces several inherent challenges that must be acknowledged. First, accessing the most relevant tissue, the foetal brain, is obviously not feasible in living humans. Therefore, studies rely on proxy tissues like placenta, umbilical cord blood, or neonatal blood spots to infer what might be happening in the brain [43,78,83]. A second major limitation is tissue specificity. Because the human foetal brain is inaccessible, researchers rely on surrogate tissues such as cord blood, placenta, or neonatal dried blood spots. While these offer important insights, epigenetic signatures are often highly tissue- and cell-type-specific, and findings in peripheral tissues may not reflect changes in neurons or glial cells. Even within the placenta or blood, cellular heterogeneity can obscure true signals unless single-cell or deconvolution methods are applied. A methylation change in cord blood immune cells may not necessarily reflect changes in neurons. The placenta, being the central mediator of maternal–foetal exchange, is a particularly informative tissue and has yielded insights (e.g., placental methylation correlating with later cognition). However, the placenta’s epigenome is unique (it undergoes extensive DNA demethylation and genomic imprinting) and not identical to the brain’s [117]. Thus, a key challenge is bridging the gap between peripheral/placental epigenetic markers and foetal brain epigenetic changes. Advanced methods, like developing organoids or using animal models to validate human findings, are employed to address this [118]. One of the most significant challenges in prenatal epigenetics is the inability to infer causality from most existing data. The majority of studies to date are observational, meaning that associations between maternal exposures and offspring methylation patterns may reflect correlation rather than causation. Even when controlling for confounders (e.g., socioeconomic status, genetics), reverse causality or unmeasured third variables cannot be ruled out. Mendelian randomisation offers one partial solution but requires large datasets and robust genetic instruments, which are not always available for prenatal exposures [25]. Maternal exposures are not random; for instance, mothers who smoke in pregnancy may differ in socioeconomic or genetic ways that also affect child outcomes [67]. Epigenetic associations could be correlative; perhaps genetic variants predispose a mother to both depression and to particular placental methylation, explaining the observed link between maternal depression and infant methylation without a true causal path. Studies try to account for confounders (like socioeconomic status, maternal diet, postnatal environment) in statistical models, but residual confounding is hard to eliminate.

Small sample sizes and lack of replication have also been significant issues. Epigenome-wide association studies (EWAS) of prenatal exposures often identify dozens of significant CpGs, but when another cohort tries to replicate, only a handful overlap. The 2023 systematic review of infant EWAS for maternal depression/anxiety found almost no single CpG reproduced across studies, highlighting low signal-to-noise ratio and possibly the transient nature of some changes. Part of this is due to sample size; many studies had <100 mother–infant pairs, underpowered to detect small effects amidst the epigenetic noise of inter-individual variability [12]. New consortia (like the PACE consortium—Pregnancy And Childhood Epigenetics) have formed to aggregate data from multiple cohorts, boosting power to detect true associations and filter out false positives [119]. As data accumulates, meta-analyses will help clarify which epigenetic changes are robust.

Another technical challenge is distinguishing epigenetic changes caused by prenatal exposure from those that are consequences of the developing disorder or other postnatal influences. For example, if one finds differential methylation in adolescents with ASD who had prenatal infection exposure, was that set in motion in utero or did it arise later because the child had autism (and maybe different life experiences or medications)? Prospective longitudinal designs, where epigenetics is measured at birth (before the disorder manifests) and again later, help address this [120]. If a methylation difference is present at birth in those who go on to develop the disorder, that strengthens the argument for prenatal origin. Few cohorts currently have long-term follow-up of neonatal epigenome into adulthood, but that is a critical direction for future research. Furthermore, cell-type heterogeneity within tissues can obscure findings. Cord blood or placenta is composed of many cell types, each with distinct epigenetic signatures. An exposure might affect one cell type strongly (say, placental trophoblasts) but if the tissue is analysed in bulk, the signal is diluted [121]. Techniques like cell-sorting or single-cell epigenomic profiling are beginning to be applied to placental tissue and cord blood to pinpoint cell-specific effects [122]. For instance, exposure might demethylate regulatory regions in foetal brain neurons, but we might only detect it if we look at neural-derived exosomes or specific foetal cell populations. This adds complexity but is increasingly feasible with new technologies.

Finally, there remains a translational gap between identifying epigenetic associations and implementing them in clinical practice. Many proposed biomarkers have not been replicated across cohorts, and effect sizes tend to be small. Moreover, the plasticity of the epigenome across development adds complexity: a methylation mark at birth may or may not persist into later life, making its predictive utility uncertain. Translating findings into interventions is further complicated by ethical, safety, and timing considerations, particularly when dealing with pregnant populations. Nonetheless, acknowledging and addressing these limitations is essential for responsibly advancing the field toward precision psychiatry and early prevention.

### 8.2. Future Directions

The coming years will likely witness major advances in integrating prenatal epigenetic insights into a more predictive and preventive framework for psychiatry and other medical conditions. One direction is developing epigenetic biomarkers of risk that can be measured at birth (or even via a prenatal biopsy like amniocentesis or cell-free foetal DNA in maternal blood). If a reproducible panel of epigenetic marks indicative of high risk for, say, autism or schizophrenia could be identified, those infants could be monitored closely or offered early interventions [123]. For example, a combination of placental DNA methylation at neurodevelopmental genes and obstetric complication data might enhance the prediction of which high-genetic-risk infants actually develop schizophrenia decades later [124]. This would be a game-changer for implementing preventive mental health measures. Another future avenue is precision medicine trials. If we know a particular prenatal pathway is disrupted (e.g., maternal inflammation causing IL-6 elevation and altered foetal brain epigenetics), we might trial specific interventions like anti-inflammatory agents in pregnant women at risk [51]. The challenge is doing this safely. There is already interest in testing whether administering certain neuroprotective nutrients (like choline, omega-3 fatty acids, or vitamin D) to pregnant women with high risk factors, such as a previous child with autism or significant infection during pregnancy, can reduce incidence of disorders [108]. As an example, a randomised trial is underway giving high-dose choline to pregnant women based on the hypothesis that it will lower schizophrenia-spectrum disorders in their offspring; outcomes will not be known for years, but if successful, it validates the prenatal nutritional epigenetic intervention approach [125].

Cross-disciplinary integration will be crucial. Epigenetics does not operate in isolation; it interacts with genetics, transcriptomics, the prenatal environment, and the postnatal caregiving [126]. Future studies will likely measure multiple layers (“multi-omics”), for instance, capturing genetic risk scores, placental transcriptomes, DNA methylation, and even foetal brain imaging [127]. A prospective study might correlate a constellation of factors (e.g., maternal cortisol, infant NR3C1 methylation, amygdala volume on neonatal MRI) to better predict anxiety risk. Integrating these data into predictive models is complex but aligns with a precision psychiatry vision where clinicians could use an array of biological and psychosocial indicators to tailor prevention.

There is also interest in how prenatal epigenetic changes might interact with later life interventions (gene–environment–epigenetic interplay over the life course) [128]. For example, if a child is born with a certain methylation profile due to prenatal stress, would that child respond differently to a positive parenting intervention at age 2 compared to a child without that profile? This is a question of moderated intervention effectiveness by early epigenetic state and researching it could help customise early childhood programs to those who need them most. Another future direction is exploring sex-specific epigenetic markers more deeply. As repeatedly noted, males and females can differ in how their epigenome responds to the same exposure [40,72]. Future studies will likely stratify or separately analyse by sex to identify male-specific and female-specific epigenetic predictors. This could lead to sex-tailored interventions (for instance, male foetuses might benefit more from certain anti-stress interventions given their higher sensitivity in some contexts).

In terms of technology, epigenomic editing tools and better epigenetic sequencing (even in single cells) will open new experimental possibilities [129]. Researchers may eventually be able to simulate human prenatal exposure by editing the epigenome of stem-cell-derived neural cells and see how it alters development, which can validate causal roles of specific epigenetic changes. Conversely, they might correct an epigenetic defect in vitro to see if a developmental phenotype is rescued, informing therapeutic target development. Finally, ethical and policy considerations will come to the forefront. If we can identify children at birth as high risk for mental illness via epigenetics, ensuring that information is used to support and not stigmatise will be essential [130]. It also raises questions about recommending interventions during pregnancy; for example, should all pregnant women in high-pollution areas receive certain supplements or air filters as a public health measure? Science may drive policy changes such as expanded maternal nutrition programs, stress reduction initiatives (e.g., making psychotherapy more available in prenatal care), and stricter environmental regulations to protect developing brains.

### 8.3. Limitations of This Review

Our review, as a narrative rather than systematic review, it is subject to selection bias in study inclusion, despite our efforts to ensure comprehensive coverage of recent high-impact findings. Many of the associations described stem from observational studies, which cannot infer causality (as thoroughly described in Section 8.1). Additionally, while we focus on human data, the reliance on surrogate tissues (e.g., placenta, cord blood) as proxies for brain epigenetic changes limits generalizability. Finally, although we prioritised recent literature (2010–2025), some emerging studies may not yet be indexed or peer-reviewed, and our synthesis inevitably reflects the current state of published evidence. These constraints should be considered when interpreting our conclusions.

## 9. Conclusions

While considerable methodological and conceptual challenges remain, the field of prenatal epigenetic research is advancing at a remarkable pace. Driven by the consolidation of interdisciplinary approaches, the increasing availability of high-resolution epigenomic data, and a growing commitment to longitudinal cohort designs, we are beginning to untangle the complex interplay between intrauterine exposures and neurodevelopmental trajectories. As the evidence base matures, so too does the promise of applying these insights to real-world contexts. The prospect of integrating epigenetic biomarkers into early-life screening and risk stratification frameworks is no longer aspirational; it is, increasingly, a feasible component of precision psychiatry. To realise this potential, however, we must continue to refine our methodological tools, particularly in relation to tissue specificity, causal inference, and temporal resolution, while fostering large-scale, multi-site collaborations capable of capturing population heterogeneity. If successful, these endeavours may allow obstetricians, paediatricians, and mental health professionals to act not only as first responders but as proactive caregivers of neurodevelopmental health. By detecting epigenetic footprints of risk before symptoms emerge, we may eventually develop preventative interventions that are not only timely but also tailored, potentially shifting the epidemiological burden of psychiatric disorders across generations. In this light, the journey from womb to mind is not merely a metaphorical arc; it is an epigenetic continuum, a dynamic, plastic, and sensitive machinery influenced by internal and external environmental cues. Mapping this continuum in finer detail represents one of the most promising avenues for advancing our understanding of mental health aetiology and, crucially, for translating that understanding into interventions that are both equitable and effective.

## Figures and Tables

**Table 1:** Summary of prenatal epigenetic mechanisms, key exposures, affected tissues, and associated outcomes.

Epigenetic Mechanism	Key Prenatal Exposures	Affected Tissue(s)	Associated Psychiatric/Neurodevelopmental Outcomes	Key References
DNA Methylation	Maternal stress, malnutrition, smoking, infections	Cord blood, placenta, neonatal blood	Schizophrenia, ASD, ADHD, depression, anxiety	[22,24,36,37,38]
Histone Modifications	Valproic acid, stress, toxins	Placenta (mostly in animal models)	ASD, cognitive impairment (suggestive in humans)	[25,28,29,30]
Non-coding RNAs	Maternal anxiety, inflammation	Placenta, foetal tissues	ASD, emotion regulation problems	[33,34,35]
Placental Gene Regulation	Stress, infection, maternal obesity	Placenta	Schizophrenia, ASD, anxiety (sex-specific effects)	[37,39,40]
Combined Mechanisms	Dutch famine, COVID-19, lead exposure	Cord blood, placenta, brain (animal)	Schizophrenia, cognitive dysfunction, ADHD	[23,41,42,43]

## Data Availability

Not applicable.
